# Supplementary material for: Systems consolidation induces multiple memory engrams for a flexible recall strategy in observational fear memory in male mice
Source: Nat Commun. 2023 Jul 5;14:3976. doi: 10.1038/s41467-023-39718-5 (PMC10322999; doi:10.1038/s41467-023-39718-5)
Supplement: Supplementary file 1 — Supplementary Information [file 41467_2023_39718_MOESM1_ESM.pdf]

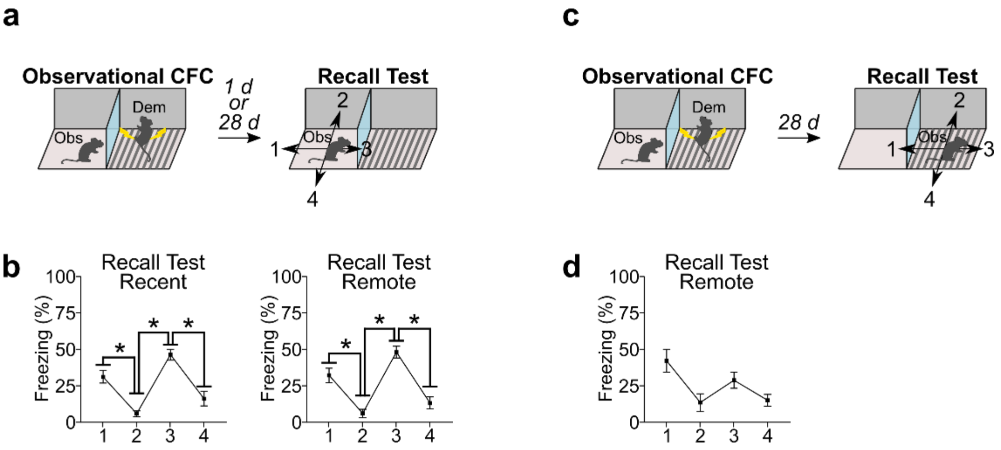

Supplemental Fig. 1. Head direction of observer during recall of observational CFC memory. **(a, c)** Head direction of observer during recall of observational CFC memory in observer chamber (a) or demonstrator chamber (c). 1; Observer head direction towards the back wall on the same side. 2; Observer head direction towards the upper wall. 3; Observer head direction towards opposite chamber. 4; Observer head direction towards the plastic window (facing the camera). **(b)** Head direction of observer during freezing episodes in recall of observational CFC memory in the observer chamber at the recent time point (left) or remote time point (right). Recent time point: $F_{(3,27)}$ = 15.22, **** P < 0.0001, BF_{incl} = 169,193.10. 1 vs. 2: ** P = 0.002. 2 vs. 3: **** P < 0.0001. 3 vs. 4: * P = 0.01. N = 10. Repeated measures One-way ANOVA with Tukey test. Remote time point: $F_{(3,27)}$ = 16.20, **** P < 0.0001, BF_{incl} = 31,807.03. N = 10. 1 vs. 2: * P = 0.01. 2 vs. 3: *** P = 0.0002. 3 vs. 4: * P = 0.02. Repeated measures One-way ANOVA with Tukey test. **(d)** Head direction of observer during freezing episodes in recall of observational CFC memory in the demonstrator chamber at the remote time point. $F_{(3,27)}$ = 3.78, * P = 0.04, BF_{incl} = 9.34. All post-hoc comparison are non-significant (P > 0.05). N = 10 Repeated measures One-way ANOVA with Tukey test. Data in b, d are presented as mean values +/- SEM. Source data are provided as a Source Data file.

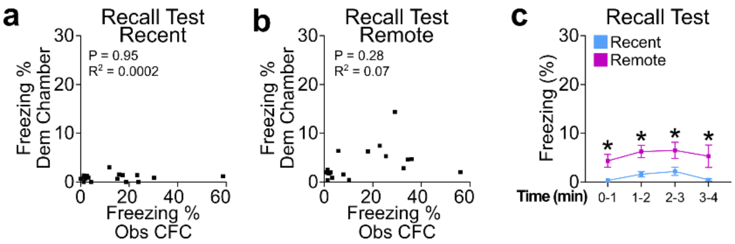

Supplemental Fig. 2. Recall of observational CFC memory in demonstrator chamber. **(a-b)** Correlation of observer freezing levels during observational CFC (or non-shock condition) with freezing levels during recall of observational CFC memory in demonstrator chamber at the recent (a) or remote (b) time points. Recent: R^2 = 0.0002, P = 0.95, BF_{10} = 0.28. N = 20. Remote: R^2 = 0.07, P = 0.28, BF_{10} = 0.49. N = 20 (1 outlier removed). Pearson's correlation coefficient (two-tailed). **(c)** Time course of observer freezing levels during recall of observational CFC memory in observer chamber at the recent or remote time points. Time Point Effect: $F_{(1,18)}$ = 15.35, ** P = 0.001, BF_{incl} = 16.03. Bin Main Effect: $F_{(3,54)}$ = 1.23, P = 0.27, BF_{incl} = 0.20. Bin * Time Point: $F_{(3,54)}$ = 0.06, P = 0.98, BF_{incl} = 0.14. Two-way Mixed ANOVA. N = 20 (Obs CFC, Recent: 10; Obs CFC Remote: 10). Data in a-b are presented as correlations. Data in c are presented as line graphs with mean values +/- SEM. Source data are provided as a Source Data file.

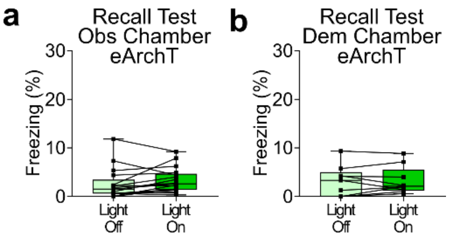

Supplemental Fig. 3. Freezing levels of observers that received Light-On + eArchT in the 2-second Light-Off period and 13-second Light-On period. **(a)** Observer chamber (from Fig. 3i). t_{16} = 1.65, P = 0.94, BF_{+0} = 0.11. N = 17. Paired t-test (one-tailed). **(b)** Demonstrator chamber (from Fig. 4i). t_8 = 0.26, P = 0.60, BF_{+0} = 0.27. N = 10 (1 outlier removed). Paired t-test (one-tailed). Graphs are presented as boxplots with minimum (lower whisker), 25th percentile (lower box bound), median (center), 75th percentile (upper box bound), and maximum (upper whisker) values indicated. Source data are provided as a Source Data file.

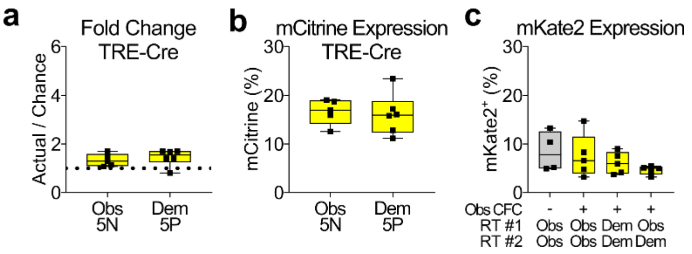

Supplemental Fig. 4. Additional analyses for activity-dependent cell labeling experiments in Figures 5 and 6. **(a)** Fold change analysis (actual / chance) for Fig. 5n and 5p. Dotted line; chance level (Fold change = 1). $t_9 = 0.64$, $P = 0.54$, $BF_{10} = 0.55$. N = 11 (5 Obs 5n; 6 Dem 5p). Unpaired t-test (two-tailed). **(b)** Expression of mCitrine$^+$ neurons in Figure 5n and 5p. $t_9 = 0.26$, $P = 0.80$, $BF_{10} = 0.49$. N = 11 (5 Obs; 6 Dem). Unpaired t-test (two-tailed). **(c)** Expression of mKate2$^+$ neurons in Figure 6. $F_{(3,15)} = 1.27$, $P = 0.32$, $BF_{incl} = 0.55$. N = 19 (Obs/Obs, Non-Shock: 4; Obs/Obs, Obs CFC: 5; Dem/Dem, Obs CFC: 5; Obs/Dem, Obs CFC: 5). One-way ANOVA. Graphs are presented as boxplots with minimum (lower whisker), 25th percentile (lower box bound), median (center), 75th percentile (upper box bound), and maximum (upper whisker) values indicated. Source data are provided as a Source Data file.

Supplemental Table 1 - Statistical Details of Experiments				
**Figure**	**Independent Variables**	**Statistical Test**	**P-Value**	**Bayesian Factor**
**1B**	N = 40<br>Non-Shock, Recent (10)<br>Obs CFC, Recent (10)<br>Non-Shock, Remote (10)<br>Obs CFC, Remote (10)	2x2 between-subjects ANOVA with Bonferroni test	Treatment: $F_{(1,36)} = 21.44$, **** $P < 0.0001$<br>Time Point: $F_{(1,36)} = 2.34$, $P = 0.14$<br>Interaction: $F_{(1,36)} = 1.42$, $P = 0.24$<br><br><u>Bonferroni test</u><br>Non-Shock Recent vs. Shock Recent:<br>* $P = 0.04$<br><br>Non-Shock Remote vs. Shock Remote:<br>*** $P = 0.0004$	Treatment: $BF_{incl} = 336.05$<br>Time Point: $BF_{incl} = 0.83$<br>Interaction: $BF_{incl} = 1.12$
**1C**	N = 20<br>Obs CFC, Recent (20)	Pearson's correlation coefficient (two-tailed)	$R^2 = 0.54$, *** $P = 0.0002$	$BF_{10} = 144.79$
**1D**	N = 20<br>Obs CFC, Remote (20)	Pearson's correlation coefficient (two-tailed)	$R^2 = 0.54$, *** $P = 0.0002$	$BF_{10} = 154.51$
**1E**	N = 20<br>Obs CFC, Recent (10)<br>Obs CFC, Remote (10)	2x2 mixed ANOVA	Bin: $F_{(3,54)} = 0.47$, $P = 0.62$<br>Time Point: $F_{(1,18)} = 1.98$, $P = 0.18$<br>Interaction: $F_{(3,54)} = 1.45$, $P = 0.24$	Bin: $BF_{incl} = 0.09$<br>Time Point: $BF_{incl} = 0.55$<br>Interaction: $BF_{incl} = 0.09$
**1G**	N = 40<br>Non-Shock, Recent (10, 1 outlier removed)<br>Obs CFC, Recent (10)<br>Non-Shock, Remote (10)<br>Obs CFC, Remote (10)	2x2 between-subjects ANOVA	Treatment: $F_{(1,35)} = 0.38$, $P = 0.54$<br>Time Point: $F_{(1,35)} = 3.26$, $P = 0.08$<br>Interaction: $F_{(1,35)} = 3.33$, $P = 0.08$	Treatment: $BF_{incl} = 0.40$<br>Time Point: $BF_{incl} = 0.90$<br>Interaction: $BF_{incl} = 0.71$

**1I**	N = 40<br>Non-Shock, Recent (10)<br>Obs CFC, Recent (10)<br>Non-Shock, Remote (10, 1 outlier removed)<br>Obs CFC, Remote (10)	2x2 between-subjects ANOVA with Bonferroni test	Treatment: $F_{(1,35)} = 13.59$, ** $P = 0.001$<br>Time Point: $F_{(1,35)} = 13.19$, ** $P = 0.001$<br>Interaction: $F_{(1,35)} = 7.39$, * $P = 0.01$<br><br><u>Bonferroni test</u><br>Non-Shock Recent vs. Shock Recent:<br>*$P = 0.98$*<br><br>Non-Shock Remote vs. Shock Remote:<br>*** $P = 0.0002$	Treatment: $BF_{incl} = 58.46$<br>Time Point: $BF_{incl} = 69.97$<br>Interaction: $BF_{incl} = 17.89$
**1K**	N = 20<br>Non-Shock (10)<br>Obs CFC (10)	Unpaired t-test (one-tailed)	$t_{18} = 1.91$, $P = 0.96$	$BF_{-0} = 0.17$
**1M**	N = 20<br>Non-Shock (10)<br>Obs CFC (10, 1 outlier removed)	Mann-Whitney U-test (one-tailed)	$U = 28.50$, $P = 0.92$	$BF_{-0} = 0.21$
**1O (left)**	N = 20<br>Non-Shock (10)<br>Obs CFC (10)	2x2 mixed ANOVA with Bonferroni test	Treatment: $F_{(1,18)} = 22.88$, *** $P = 0.0001$<br>Chamber: $F_{(1,18)} = 24.02$, *** $P = 0.0001$<br>Interaction: $F_{(1,18)} = 27.00$,<br>**** $P < 0.0001$<br><br><u>Bonferroni test</u><br>Non-Shock Obs Chamber Freezing vs. Obs CFC Obs Chamber Freezing: **** $P < 0.0001$<br><br>Non-Shock Dem Chamber Freezing vs. Obs CFC Dem Chamber Freezing:<br>$P > 0.99$	Treatment: $BF_{incl} = 26{,}196.16$<br>Chamber: $BF_{incl} = 90{,}495.41$<br>Interaction: $BF_{incl} = 9{,}975.29$
**1O (right)**	N = 20<br>Non-Shock (10)<br>Obs CFC (10)	Unpaired t-test (two-tailed)	$t_{18} = 0.49$, $P = 0.63$	$BF_{10} = 0.43$

**1P (left)**	N = 24<br>Non-Shock (12)<br>Obs CFC (12)	2x2 mixed ANOVA<br>with Bonferroni test	Treatment: $F_{(1,22)}$ = 11.72, ** P = 0.002<br>Chamber: $F_{(1,22)}$ = 3.39, P = 0.08<br>Interaction: $F_{(1,22)}$ = 0.51, P = 0.48<br><br>Bonferroni test<br>Non-Shock Obs Chamber Freezing vs. Obs CFC Obs Chamber Freezing: ** P = 0.004<br><br>Non-Shock Dem Chamber Freezing vs. Obs CFC Dem Chamber Freezing:<br>* P = 0.030	Treatment: BF_{incl} = 12.17<br>Chamber: BF_{incl} = 1.00<br>Interaction: BF_{incl} = 0.91
**1P (right)**	N = 24<br>Non-Shock (12)<br>Obs CFC (12)	Unpaired t-test (two-tailed)	t_{22} = 2.35, * P = 0.03	BF_{10} = 2.46
**2D**	N = 20<br>Non-Shock, Recent (5)<br>Obs CFC, Recent (5)<br>Non-Shock, Remote (5)<br>Obs CFC, Remote (5)	2x2 between-subjects ANOVA<br>with Bonferroni test	Treatment: $F_{(1,16)}$ = 1.96, P = 0.18<br>Time Point: $F_{(1,16)}$ = 18.04, ** P = 0.001<br>Interaction: $F_{(1,16)}$ = 7.22, * P = 0.02<br><br>Bonferroni test<br>Non-Shock Recent vs. Obs CFC Recent: * P = 0.02<br><br>Non-Shock Remote vs. Obs CFC Remote: P = 0.75	Treatment: BF_{incl} = 1.77<br>Time Point: BF_{incl} = 27.60<br>Interaction: BF_{incl} = 4.94

**2G**	N = 20<br>Non-Shock, Recent (5)<br>Obs CFC, Recent (5)<br>Non-Shock, Remote (5)<br>Obs CFC, Remote (5)	2x2 between-subjects ANOVA with Bonferroni test	Treatment: $F_{(1,16)} = 1.07$, $P = 0.32$<br>Time Point: $F_{(1,16)} = 3.77$, $P = 0.07$<br>Interaction: $F_{(1,16)} = 7.84$, * $P = 0.01$<br><br><u>Bonferroni test</u><br>Non-Shock Recent vs. Obs CFC Recent: * $P = 0.03$<br><br>Non-Shock Remote vs. Obs CFC Remote: $P = 0.46$	Treatment: $BF_{incl} = 1.14$<br>Time Point: $BF_{incl} = 1.73$<br>Interaction: $BF_{incl} = 3.14$
**2K**	N = 41<br>mCherry, Recent (10)<br>hM4Di, Recent (11, 1 outlier removed)<br>mCherry, Remote (10)<br>hM4Di, Remote (10)	2x2 between-subjects ANOVA with Bonferroni test	Treatment: $F_{(1,36)} = 10.52$, ** $P = 0.003$<br>Time Point: $F_{(1,36)} = 1.42$, $P = 0.24$<br>Interaction: $F_{(1,36)} = 4.93$, * $P = 0.03$<br><br><u>Bonferroni test</u><br>mCherry Recent vs. hM4Di Recent: ** $P = 0.001$<br><br>mCherry Remote vs. hM4Di Remote: $P = 0.95$	Treatment: $BF_{incl} = 12.33$<br>Time Point: $BF_{incl} = 1.00$<br>Interaction: $BF_{incl} = 2.75$
**3C**	N = 20<br>Non-Shock, Recent (5)<br>Obs CFC, Recent (5)<br>Non-Shock, Remote (5)<br>Obs CFC, Remote (5)	2x2 between-subjects ANOVA with Bonferroni test	Treatment: $F_{(1,16)} = 26.52$, **** $P < 0.0001$<br>Time Point: $F_{(1,16)} = 4.43$, $P = 0.05$<br>Interaction: $F_{(1,16)} = 8.34$, * $P = 0.01$<br><br><u>Bonferroni test</u><br>Non-Shock Recent vs. Obs CFC Recent: $P = 0.26$<br><br>Non-Shock Remote vs. Obs CFC Remote: **** $P < 0.0001$	Treatment: $BF_{incl} = 100.30$<br>Time Point: $BF_{incl} = 3.56$<br>Interaction: $BF_{incl} = 8.26$

**3D**	N = 20<br>Non-Shock, Recent (5)<br>Obs CFC, Recent (5)<br>Non-Shock, Remote (5)<br>Obs CFC, Remote (5)	2x2 between-subjects ANOVA with Bonferroni test	Treatment: $F_{(1,16)} = 10.91$, ** $P = 0.004$<br>Time Point: $F_{(1,16)} = 0.34$, $P = 0.57$<br>Interaction: $F_{(1,16)} = 10.54$, * $P = 0.01$<br><br><u>Bonferroni test</u><br>Non-Shock Recent vs. Obs CFC Recent: $P > 0.99$<br><br>Non-Shock Remote vs. Obs CFC Remote: ** $P = 0.001$	Treatment: BFincl = 8.87<br>Time Point: BFincl = 2.07<br>Interaction: BFincl = 7.56
**3E**	N = 20<br>Non-Shock, Recent (5)<br>Obs CFC, Recent (5)<br>Non-Shock, Remote (5)<br>Obs CFC, Remote (5)	2x2 between-subjects ANOVA with Bonferroni test	Treatment: $F_{(1,16)} = 30.16$, **** $P < 0.0001$<br>Time Point: $F_{(1,16)} = 3.48$, $P = 0.08$<br>Interaction: $F_{(1,16)} = 8.05$, * $P = 0.01$<br><br><u>Bonferroni test</u><br>Non-Shock Recent vs. Obs CFC Recent: $P = 0.16$<br><br>Non-Shock Remote vs. Obs CFC Remote: **** $P < 0.00001$	Treatment: $BF_{incl} = 165.33$<br>Time Point: $BF_{incl} = 2.77$<br>Interaction: $BF_{incl} = 6.97$
**3F**	N = 20<br>Non-Shock, Recent (5)<br>Obs CFC, Recent (5)<br>Non-Shock, Remote (5)<br>Obs CFC, Remote (5)	2x2 between-subjects ANOVA	Treatment: $F_{(1,16)} = 2.70$, $P = 0.12$<br>Time Point: $F_{(1,16)} = 3.54$, $P = 0.08$<br>Interaction: $F_{(1,16)} = 0.94$, $P = 0.35$	Treatment: $BF_{incl} = 0.86$<br>Time Point: $BF_{incl} = 1.10$<br>Interaction: $BF_{incl} = 0.72$
**3I**	N = 52<br>Light Off (LOFF), eYFP (9)<br>Light Off (LOFF), eArchT (10)<br>Light On (LON), eYFP (16)<br>Light On (LON), eArchT (17)	2x2 between-subjects ANOVA with Tukey test	Treatment: $F_{(1,48)} = 1.49$, $P = 0.23$<br>Light: $F_{(1,48)} = 7.40$, * $P = 0.01$<br>Interaction: $F_{(1,48)} = 4.67$, * $P = 0.04$<br><br><u>Tukey test</u><br>LOFF/eYFP vs. LOFF/eArchT: $P = 0.93$<br>LOFF/eYFP vs. LON/eYFP: $P = 0.98$<br>LOFF/eYFP vs. LON/eArchT: * $P = 0.04$<br>LOFF/eArchT vs. LON/eYFP: $P = 0.71$<br>LOFF/eArchT vs. LON/eArchT: * $P = 0.01$<br>LON/eYFP vs. LON/eArchT: * $P = 0.04$	Treatment: $BF_{incl} = 2.03$<br>Light: $BF_{incl} = 7.25$<br>Interaction: $BF_{incl} = 4.01$

**3J**	N = 22<br>Light Off (LOFF), eYFP (5)<br>Light Off (LOFF), eArchT (5)<br>Light On (LON), eYFP (6)<br>Light On (LON), eArchT (6)	2x2 between-subjects ANOVA with Tukey test	Treatment: $F_{(1,18)} = 6.15$, * $P = 0.02$<br>Light: $F_{(1,18)} = 36.18$, **** $P < 0.0001$<br>Interaction: $F_{(1,18)} = 11.24$, ** $P = 0.004$<br><br>Tukey test<br>LOFF/eYFP vs. LOFF/eArchT: $P > 0.99$<br>LOFF/eYFP vs. LON/eYFP: $P = 0.46$<br>LOFF/eYFP vs. LON/eArchT:<br>**** $P < 0.0001$<br>LOFF/eArchT vs. LON/eYFP: $P = 0.13$<br>LOFF/eArchT vs. LON/eArchT:<br>**** $P < 0.0001$<br>LON/eYFP vs. LON/eArchT: ** $P = 0.002$	Treatment: $BF_{incl} = 12.16$<br>Light: $BF_{incl} = 611.06$<br>Interaction: $BF_{incl} = 22.45$
**4B**	N = 23<br>Non-Shock, Recent (5)<br>Obs CFC, Recent (5)<br>Non-Shock, Remote (7)<br>Obs CFC, Remote (6)	2x2 between-subjects ANOVA with Bonferroni test	Treatment: $F_{(1,19)} = 0.04$, $P = 0.84$<br>Time Point: $F_{(1,19)} = 1.32$, $P = 0.26$<br>Interaction: $F_{(1,19)} = 6.28$, * $P = 0.02$<br><br>Bonferroni test<br>Non-Shock Recent vs. Obs CFC Recent:<br>$P = 0.17$<br><br>Non-Shock Remote vs. Obs CFC Remote:<br>$P = 0.20$	Treatment: $BF_{incl} = 0.58$<br>Time Point: $BF_{incl} = 0.77$<br>Interaction: $BF_{incl} = 1.40$
**4C**	N = 22<br>Non-Shock, Recent (5)<br>Obs CFC, Recent (5)<br>Non-Shock, Remote (7)<br>Obs CFC, Remote (5)	2x2 between-subjects ANOVA with Bonferroni test	Treatment: $F_{(1,18)} = 0.16$, $P = 0.70$<br>Time Point: $F_{(1,18)} = 0.77$, $P = 0.39$<br>Interaction: $F_{(1,18)} = 2.72$, $P = 0.12$	Treatment: $BF_{incl} = 0.37$<br>Time Point: $BF_{incl} = 0.43$<br>Interaction: $BF_{incl} = 0.45$

**4D**	N = 20<br>Non-Shock, Recent (5)<br>Obs CFC, Recent (5)<br>Non-Shock, Remote (5)<br>Obs CFC, Remote (5)	2x2 between-subjects ANOVA with Bonferroni test	Treatment: $F_{(1,16)} = 0.61$, $P = 0.45$<br>Time Point: $F_{(1,16)} = 10.64$, * $P = 0.01$<br>Interaction: $F_{(1,16)} = 4.27$, $P = 0.06$<br><br>Bonferroni test<br>Non-Shock Recent vs. Obs CFC Recent:<br>P = 0.75<br><br>Non-Shock Remote vs. Obs CFC Remote:<br>P = 0.12	Treatment: $BF_{incl} = 0.77$<br>Time Point: $BF_{incl} = 6.84$<br>Interaction: $BF_{incl} = 1.85$
**4E**	N = 20<br>Non-Shock, Recent (5)<br>Obs CFC, Recent (5)<br>Non-Shock, Remote (5)<br>Obs CFC, Remote (5)	2x2 between-subjects ANOVA with Bonferroni test	Treatment: $F_{(1,16)} = 3.33$, $P = 0.09$<br>Time Point: $F_{(1,16)} = 6.86$, * $P = 0.02$<br>Interaction: $F_{(1,16)} = 1.98$, $P = 0.18$<br><br>Bonferroni test<br>Non-Shock Recent vs. Obs CFC Recent:<br>$P > 0.99$<br><br>Non-Shock Remote vs. Obs CFC Remote:<br>$P = 0.07$	Treatment: $BF_{incl} = 1.18$<br>Time Point: $BF_{incl} = 2.80$<br>Interaction: $BF_{incl} = 1.37$
**4F**	N = 20<br>Non-Shock, Recent (5)<br>Obs CFC, Recent (5)<br>Non-Shock, Remote (5)<br>Obs CFC, Remote (5)	2x2 between-subjects ANOVA with Bonferroni test	Treatment: $F_{(1,16)} = 16.33$, ** $P = 0.001$<br>Time Point: $F_{(1,16)} = 23.67$, *** $P = 0.0002$<br>Interaction: $F_{(1,16)} = 7.25$, * $P = 0.02$<br><br>Bonferroni test<br>Non-Shock Recent vs. Obs CFC Recent:<br>$P = 0.71$<br><br>Non-Shock Remote vs. Obs CFC Remote:<br>*** $P = 0.0004$	Treatment: $BF_{incl} = 26.50$<br>Time Point: $BF_{incl} = 74.78$<br>Interaction: $BF_{incl} = 10.84$

**4G**	N = 20<br>Non-Shock, Recent (5)<br>Obs CFC, Recent (5)<br>Non-Shock, Remote (5)<br>Obs CFC, Remote (5)	2x2 between-subjects ANOVA<br>with Bonferroni test	Treatment: $F_{(1,16)}$ = 0.01, *P* = 0.91<br>Time Point: $F_{(1,16)}$ = 11.24, ** *P* = 0.004<br>Interaction: $F_{(1,16)}$ = 8.13, * *P* = 0.01<br><br><u>Bonferroni test</u><br>Non-Shock Recent vs. Obs CFC Recent:<br>*P* = 0.14<br><br>Non-Shock Remote vs. Obs CFC Remote:<br>*P* = 0.10	Treatment: BF_{incl} = 1.23<br>Time Point: BF_{incl} = 7.92<br>Interaction: BF_{incl} = 4.14
**4I**	N = 20<br>Non-Shock (10)<br>Obs CFC (10, 1 outlier removed)	Unpaired t-test (one-tailed)	t_{17} = 2.76, * *P* = 0.01	BF_{+0} = 8.29
**5B**	N = 10<br>Non-Shock (5)<br>Obs CFC (5)	Unpaired t-test (two-tailed)	t_8 = 2.20, *P* = 0.06	BF_{10} = 1.68
**5C**	N = 10<br>Non-Shock (5)<br>Obs CFC (5)	Unpaired t-test (two-tailed)	t_8 = 2.49, * *P* = 0.04	BF_{10} = 2.21
**5D**	N = 10<br>Non-Shock (5)<br>Obs CFC (5)	Unpaired t-test (two-tailed)	t_8 = 4.14, ** *P* = 0.003	BF_{10} = 11.39
**5E**	N = 10<br>Non-Shock (5)<br>Obs CFC (5)	Mann Whitney U-test (two-tailed)	*U* = 8, *P* = 0.42	BF_{10} = 0.61
**5J**	N = 22<br>mCherry (11, 1 outlier removed)<br>hM4Di (11)	2x2 mixed ANOVA<br>with Bonferroni test	Treatment: $F_{(1,19)}$ = 1.13, *P* = 0.30<br>Time Point: $F_{(1,19)}$ = 4.30, *P* = 0.05<br>Interaction: $F_{(1,19)}$ = 5.75, * *P* = 0.03<br><br><u>Bonferroni test</u><br>mCherry Recent vs. hM4Di Recent:<br>*P* = 0.75<br><br>mCherry Remote vs. hM4Di Remote:<br>* *P* = 0.04	Treatment: BF_{incl} = 1.21<br>Time Point: BF_{incl} = 2.50<br>Interaction: BF_{incl} = 3.56

**5K**	N = 21<br>mCherry (10)<br>hM4Di (11, 1 outlier removed)	2x2 mixed ANOVA<br>with Bonferroni test	Treatment: $F_{(1,18)} = 9.70$, * $P = 0.01$<br>Time Point: $F_{(1,18)} = 19.85$, *** $P = 0.0003$<br>Interaction: $F_{(1,18)} = 8.23$, * $P = 0.01$<br><br><u>Bonferroni test</u><br>mCherry Recent vs. hM4Di Recent:<br>$P > 0.99$<br><br>mCherry Remote vs. hM4Di Remote:<br>*** $P = 0.0003$	Treatment: $BF_{incl} = 25.11$<br>Time Point: $BF_{incl} = 928.73$<br>Interaction: $BF_{incl} = 31.86$
**5N**	N = 5	Paired t-test (two-tailed)	$t_4 = 3.68$, * $P = 0.02$	$BF_{10} = 4.11$
**5P**	N = 6	Paired t-test (two-tailed)	$t_5 = 2.85$, * $P = 0.04$	$BF_{10} = 2.67$
**6C**	N = 4	Paired t-test (two-tailed)	$t_3 = 0.15$, $P = 0.89$	$BF_{10} = 0.43$
**6D**	N = 5	Paired t-test (two-tailed)	$t_4 = 3.44$, * $P = 0.03$	$BF_{10} = 3.50$
**6E**	N = 5	Paired t-test (two-tailed)	$t_4 = 4.47$, * $P = 0.01$	$BF_{10} = 6.54$
**6F**	N = 5	Paired t-test (two-tailed)	$t_4 = 3.44$, * $P = 0.03$	$BF_{10} = 3.52$
**6G**	N = 19<br>Obs/Obs, Non-Shock (4)<br>Obs/Obs (5)<br>Dem/Dem (5)<br>Obs/Dem (5)	One-way ANOVA with Tukey test	$F_{(3,15)} = 5.93$, * $P = 0.01$<br><br><u>Tukey test</u><br>Obs/Obs Non-Shock vs. Obs/Obs: * $P = 0.04$<br>Obs/Obs Non-Shock vs. Dem/Dem: P = 0.10<br>Obs/Obs Non-Shock vs. Obs/Dem: P = 0.99<br>Obs/Obs vs. Dem/Dem: P = 0.97<br>Obs/Obs vs. Obs/Dem: * $P = 0.02$<br>Dem/Dem vs. Obs/Dem: * $P = 0.04$	$BF_{10} = 7.70$
