# Supplementary material for: Systems consolidation induces multiple memory engrams for a flexible recall strategy in observational fear memory in male mice
Source: Nat Commun. 2023 Jul 5;14:3976. doi: 10.1038/s41467-023-39718-5 (PMC10322999; doi:10.1038/s41467-023-39718-5)
Supplement: Supplementary file 3 — Reporting Summary [file 41467_2023_39718_MOESM3_ESM.pdf]

nature portfolio

Corresponding author(s): Takashi Kitamura

Last updated by author(s): May 30, 2023

Reporting Summary

Nature Portfolio wishes to improve the reproducibility of the work that we publish. This form provides structure for consistency and transparency in reporting. For further information on Nature Portfolio policies, see our Editorial Policies and the Editorial Policy Checklist.

Statistics

For all statistical analyses, confirm that the following items are present in the figure legend, table legend, main text, or Methods section.

n/a	Confirmed	
☐	☒	The exact sample size (n) for each experimental group/condition, given as a discrete number and unit of measurement
☐	☒	A statement on whether measurements were taken from distinct samples or whether the same sample was measured repeatedly
☐	☒	The statistical test(s) used AND whether they are one- or two-sided<br>*Only common tests should be described solely by name; describe more complex techniques in the Methods section.*
☒	☐	A description of all covariates tested
☐	☒	A description of any assumptions or corrections, such as tests of normality and adjustment for multiple comparisons
☐	☒	A full description of the statistical parameters including central tendency (e.g. means) or other basic estimates (e.g. regression coefficient) AND variation (e.g. standard deviation) or associated estimates of uncertainty (e.g. confidence intervals)
☐	☒	For null hypothesis testing, the test statistic (e.g. F, t, r) with confidence intervals, effect sizes, degrees of freedom and P value noted<br>*Give P values as exact values whenever suitable.*
☐	☒	For Bayesian analysis, information on the choice of priors and Markov chain Monte Carlo settings
☒	☐	For hierarchical and complex designs, identification of the appropriate level for tests and full reporting of outcomes
☒	☐	Estimates of effect sizes (e.g. Cohen's d, Pearson's r), indicating how they were calculated

Our web collection on statistics for biologists contains articles on many of the points above.

Software and code

Policy information about availability of computer code

Data collection	Video Freeze (Med Associates, version 2.7.3), Behavioral Observation Research Interactive Software (BORIS, Friard & Gamba, version 7.13.9), Zen Blue (Zeiss, version 2.3), ImageJ (NIH, version 1.51w)
Data analysis	Prism (Graphpad, version 9.5.1), JASP (JASP Team, version 0.17.1)

For manuscripts utilizing custom algorithms or software that are central to the research but not yet described in published literature, software must be made available to editors and reviewers. We strongly encourage code deposition in a community repository (e.g. GitHub). See the Nature Portfolio guidelines for submitting code & software for further information.

Data

Policy information about availability of data

All manuscripts must include a data availability statement. This statement should provide the following information, where applicable:

- Accession codes, unique identifiers, or web links for publicly available datasets
- A description of any restrictions on data availability
- For clinical datasets or third party data, please ensure that the statement adheres to our policy

Requests for materials and correspondence should be made to the lead author, Takashi Kitamura (takashi.kitamura@utsouthwestern.edu). The datasets generated by the current study are available from the corresponding author upon request. Source Data and statistical analyses are provided with this paper.

Research involving human participants, their data, or biological material

Policy information about studies with human participants or human data. See also policy information about sex, gender (identity/presentation), and sexual orientation and race, ethnicity and racism.

Reporting on sex and gender	N/A
Reporting on race, ethnicity, or other socially relevant groupings	N/A
Population characteristics	N/A
Recruitment	N/A
Ethics oversight	N/A

Note that full information on the approval of the study protocol must also be provided in the manuscript.

Field-specific reporting

Please select the one below that is the best fit for your research. If you are not sure, read the appropriate sections before making your selection.

☒ Life sciences ☐ Behavioural & social sciences ☐ Ecological, evolutionary & environmental sciences

For a reference copy of the document with all sections, see nature.com/documents/nr-reporting-summary-flat.pdf

Life sciences study design

All studies must disclose on these points even when the disclosure is negative.

Sample size	Statistical methods were not used to predetermine sample sizes in experiments; sample sizes were selected based on what is conventional for the field, which previous studies determined were sufficiently powerful to detect meaningful differences (or lack of differences) between groups (see references 5, 9, 21, and 99).
Data exclusions	Outliers were detected using the Grubbs' method with threshold for removal set to Alpha = 0.01. Removal of any outliers is specified in Supplemental Table 1. Exclusion criteria were pre-established.
Replication	All experiments in the manuscript were conducted in a highly controlled and careful manner to ensure reproducibility and experimental rigor. Experiments were performed twice to ensure that the results were replicable, and the results were then pooled. All attempts to verify reproducibility of the findings reported in the manuscript were successful.
Randomization	Mice were randomly assigned as observers or demonstrators. Observer mice were randomly assigned to different groups.
Blinding	Experimenters were blinded to group allocation during data analysis.

Reporting for specific materials, systems and methods

We require information from authors about some types of materials, experimental systems and methods used in many studies. Here, indicate whether each material, system or method listed is relevant to your study. If you are not sure if a list item applies to your research, read the appropriate section before selecting a response.

Materials & experimental systems

n/a	Involved in the study	
☐	☒	Antibodies
☒	☐	Eukaryotic cell lines
☒	☐	Palaeontology and archaeology
☐	☒	Animals and other organisms
☒	☐	Clinical data
☒	☐	Dual use research of concern
☒	☐	Plants

Methods

n/a	Involved in the study	
☒	☐	ChIP-seq
☒	☐	Flow cytometry
☒	☐	MRI-based neuroimaging

Antibodies

Antibodies used	Anti-Arc (Synaptic Systems, 156003); Anti-NeuN (Millipore Sigma, MAB377, clone A60); AlexaFluor 488 goat anti-rabbit IgG (ThermoFisher, A11008), AlexFluor 546 goat anti-rabbit IgG (ThermoFisher, A11010), or AlexaFluor 633 goat anti-mouse IgG (ThermoFisher, A21050)
Validation	Anti-Arc (https://sys.com/product/156003), Anti-NeuN (https://www.emdmillipore.com/US/en/product/Anti-NeuN-Antibody-clone-A60,MM_NF-MAB377), AlexaFluor 488 goat anti-rabbit IgG (https://www.thermofisher.com/antibody/product/Goat-anti-Rabbit-IgG-H-L-Cross-Adsorbed-Secondary-Antibody-Polyclonal/A-11008), AlexaFluor 546 goat anti-rabbit IgG (https://www.thermofisher.com/antibody/product/Goat-anti-Rabbit-IgG-H-L-Cross-Adsorbed-Secondary-Antibody-Polyclonal/A-11010), and AlexaFluor 633 goat anti-mouse IgG (https://www.thermofisher.com/antibody/product/Goat-anti-Mouse-IgG-H-L-Cross-Adsorbed-Secondary-Antibody-Polyclonal/A-21050) are wall-validated antibodies to use in immunohistochemical staining in mice, as noted on the manufactures' websites. We have internally validated these antibodies, and used them extensively and successfully in our recently published manuscript (Terranova et al., Neuron, 2022).

Animals and other research organisms

Policy information about studies involving animals; ARRIVE guidelines recommended for reporting animal research, and Sex and Gender in Research

Laboratory animals	Mice (mus musculus), C57 B6/J & TRE-Cre, 9 to 20 weeks of age. Mice were housed in conventional facilities with an average ambient temperature of 21.11 degrees Celsius and an average humidity of 50%
Wild animals	The study did not involve wild animals.
Reporting on sex	The current study was only conducted using male mice, and the method for assigning sex was based on the external genitalia. We are planning to conduct a future study that will directly examine sex differences in observational CFC.
Field-collected samples	The study did not collect samples from the field.
Ethics oversight	All procedures relating to experimental treatments and mouse care conformed to NIH and institutinoal guidelines, and were conducted with the approval of the UT Southwestern Institutional Care and Use Committee (IACUC).

Note that full information on the approval of the study protocol must also be provided in the manuscript.
